# The Financialization of Healthcare in France: Trends and implications

**DOI:** 10.1016/j.puhip.2025.100620

**Published:** 2025-05-09

**Authors:** Benjamin Marchandot, Olivier Morel

**Affiliations:** a Research Unit - UR3074, Translational Cardiovascular Medicine, University of Strasbourg, France; b Department of Cardiology, University Hospital of Strasbourg, Strasbourg, France; c HEC Paris, Business School and Grande Ecole, Jouy-en-Josas, France; d Hanoï Medical University, Hanoï, Viet Nam

**Keywords:** Financialization, France, Health care economics, Capital

## Abstract

The healthcare system in France, once celebrated for its universal coverage and accessibility, now grapples with profound transformations driven by corporatization, polarization, and financialization. Initially founded on principles of solidarity and government support, the system provided ample opportunities for doctors to practice either in public hospitals or private settings, with fees regulated to ensure affordability. However, recent decades have seen a shift towards agreements that allow specialists to charge additional fees beyond standard rates, which are covered by private insurance or paid directly by patients. The landscape is further complicated by demographic shifts such as an aging population and rising incidences of chronic diseases, exacerbating healthcare demand while the supply of medical professionals stagnates. Urbanization has concentrated medical services, leading to dominant practices in certain specialties and longer waiting times, especially in rural areas. Financialization has emerged as a pivotal force, with private investors increasingly influencing healthcare delivery. This trend is evident in sectors like medical biology and radiology, where consolidation and profit maximization strategies may prevail, potentially compromising care quality and access. While financial influx may temporarily address funding gaps, it also risks eroding professional autonomy and patient care standards. These developments mark a schism from traditional values of the French healthcare as a public good, raising concerns about equity, regulation, and the ethical implications of intertwining medical practice with financial imperatives in France.

## 1 Introduction

In 2000, France's healthcare system was acclaimed as the world's best by the World Health Organization [1]. Yet, over two decades later, it faces a series of crises, each more severe than the last. Rooted in principles of universal coverage, solidarity, and accessibility, France's healthcare system historically relies on government coverage, complemented by private insurance. However, the landscape is rapidly evolving.

## 2 Understanding the complex dynamics of financialization and fragmentation in French healthcare

In contemporary discussions, the term “financialization” as applied to the French healthcare system, has emerged as a novel yet frequently misinterpreted concept. In many reports and media, this term has been narrowly confined to its financial connotations, invoking concerns about profit-driven motives among healthcare providers, corporate greed, the corporatization and privatization of care, and to some extend to societal polarization. A nuanced and detailed understanding of the implications of the concepts is essential to avoid an oversimplified and fear-driven narrative that fails to capture the full complexity of the recent changes facing the French healthcare model.

Let's first replace numbers with letters, because while it is important to learn to count, it is necessary to know what counts. Financialization is a term full of numbers, that counts. Financialization refers to the increasing importance of financial motives, financial markets, financial actors, and financial institutions in shaping both economy and society [2,3]. It is crucial to distinguish between financialization and privatization (Table 1). Privatization involves the transfer of ownership of segments of the healthcare system from public to private actors. Applying to the healthcare sector, financialization encompasses the inflating influence of financial actors, including commercial and investment banks, private equity firms, venture capital firms, and other types of investors, in shifting the dynamic of trade, service and commodity production associated with the healthcare model through financial strategies and mechanisms. Corporatization refers to the growing influence of large organizations that leverage market power for profit maximization. Meanwhile, polarization in healthcare refers to the increasing divisions and stark contrasts between different groups and perspectives within the healthcare system. To fully understand the recent process of commercialization and fragmentation in the French healthcare system, it is essential to briefly examine the dynamics among system structure, medical demographics, and systemic changes; developments increasingly driven by the aspirations for a better work-life balance among current and future physicians, alongside the emerging influence of financialization.

**Table 1:** Key concepts in the financialization of healthcare.

Concept	Definition	Associated Concepts
Financialization	Progressive integration of financial motives, market dynamics, and investor interests into healthcare systems, often resulting in the prioritization of profit generation over patient-centered outcomes.	Privatization, commercialization, marketization, shareholder value, profit maximization
Financial Actors	Entities such as hedge funds, private equity firms, insurers, and investment banks involved in financing and shaping healthcare policy and delivery.	Hedge funds, venture capital, private equity, insurers, financial intermediaries
Commodification	Transformation of healthcare services and products into commodities that can be bought and sold, shifting emphasis from patient needs to market value.	Market-based pricing, consumerism, service monetization, value extraction
Marketization	Expansion of market mechanisms into healthcare delivery, increasing competition and economic efficiency, but potentially eroding equity and access.	Competitive tendering, deregulation, market incentives
Privatization	Transfer of healthcare provision or financing from public to private entities, potentially leading to disparities in access and service fragmentation.	Outsourcing, public-private partnerships, user fees
Polarization	Growing disparities in access, quality, and outcomes between different socioeconomic groups due to financialized healthcare systems.	Health inequities, social gradients, underserved populations
Corporatization	Transformation of healthcare institutions into corporate entities governed by business logic and administrative hierarchies.	Corporate governance, standardization, branding
Commercialization of care	Process by which healthcare delivery becomes oriented toward revenue generation and market competition, often emphasizing profitability over equity or clinical need.	Profit incentives, patient-as-consumer model, branding,

## 3 An idyllic board game at glance

Each player begins with a robust educational foundation, having completed medical school, residency, and a fellowship. In France, no student loans are needed, so new doctors can embark on their careers unencumbered by debt. They can choose to work in public hospitals as salaried employees of the French Ministry of Health or enter private practice (Table 2). In the private sector, an increasing majority of specialists now operate under Sector 2 agreements with the public health insurance system, allowing them to charge fees above standard rates (57.4 % in 2022 vs. 38.0 % in 2003) (Table 3). Reimbursements remain tied to standard rates, leaving individual private insurers or patients to cover extra fees. Conversely, most general practitioners operate under Sector 1 agreements, adhering to fees set by the national health insurance and not charging above these rates.

**Table 2:** The French health system at a glance.

Parameter	Brief Description
**Overall Structure**	- Type: Mixed system with structural roots in social health insurance (SHI) but strong state involvement and tax-based revenue. -Coverage: Statutory health insurance covers nearly 100 % of residents. -Administration: Centralized with some regional deconcentration via the regional health agencies (ARS).
**Governance & Regulation**	-Actors: Parliament, government (Ministry of Health) and SHI jointly share leadership. -Planning: The Ministry of Health sets policy. The ARS manage health needs and resource planning at a regional level.
**Financing & Expenditure**	-Main Sources: Employer and employee payroll contributions (∼33 %), general social contributions (∼24 %), and other taxes (notably ∼20 % from Value-Added Tax). -Public Share: About 79 % of health spending is publicly funded (mainly SHI). -Spending: 12.2 % of GDP (2020), among the highest in Europe. -Cost Sharing: Required for most services, though about 96 % of the population purchases complementary private insurance (CHI) to cover co-payments.
**Service Delivery**	-Primary Care: Mostly provided by self-employed general practionners (GPs) and specialists; the system is still somewhat hospital-centric. -Hospital Care: Public, private for-profit, and private non-profit hospitals compete. -Activity-based payments -Pharmaceuticals: High consumption; efforts to boost generics uptake and reduce overprescribing.
**Coverage & Access**	-Benefits Basket: Comprehensive, defined through national positive lists. -Equity Measures: Chronic patients and low-income populations receive cost-sharing protections -Persistent geographic disparities in physician distribution and hospital capacity.
**Health Workforce**	-Physicians: Relatively low density of physicians compared to EU average, especially GPs (declining supply). -Nurses: High number of practicing nurses, though roles (and autonomy) remain narrower than in many countries. -Challenges: Unequal geographic distribution, workforce ageing, and staff retention (especially in rural areas).
**Performance & Outcomes**	-Strengths: Low mortality from treatable causes, strong universal coverage, relatively high patient satisfaction in hospitals. -Weaknesses: Higher unmet needs in some areas (“medical deserts”), high pharmaceutical spending.
**Key numbers**	-Population Coverage: 100 % coverage under statutory health insurance. 96 % of residents also carry private complementary insurance -Health Spending: Health expenditure at 12.2 % of GDP (2020)/79 % of total spending publicly financed (SHI + state), Among the highest health spending share in Europe -Health Workforce: ∼318 practising physicians per 100 000 inhabitants (2020). 1134 practising nurses per 100 000 inhabitants (2020). Unequal geographical distribution persists -Hospital Sector: ∼3000 hospitals total (45 % public, 33 % private for-profit, 22 % private non-profit). 300 acute-care beds per 100 000 inhabitants (2019). Growing emphasis on outpatient and day-case procedures -Health Outcomes: Life expectancy at 82.5 years (2021) Infant mortality: 3.8 per 1000 live births. Mortality from treatable causes: 62.1 per 100 000 (2017)

**Table 3:** Percentage of French private practice physicians allowed to charge extra fees (formerly “sector 2”), by specialty (2010–2023).

Medical Speciality	Sector 2 Proportion (2010)	Sector 2 Proportion (2023)	% Change (2023 vs 2010)
Radiologists	13,8 %	32,0 %	132,1 %
Anaesthesiologists	34,6 %	67,6 %	95,2 %
Otolaryngologists	39,8 %	71,1 %	78,6 %
Paediatricians	33,7 %	53,1 %	57,5 %
Psychiatrists	30,0 %	47,0 %	56,3 %
Cardiologists	19,9 %	29,3 %	47,4 %
Gynaecologists	55,3 %	75,5 %	36,5 %
Dermatologists	42,1 %	49,4 %	17,5 %
Surgeons	78,1 %	85,9 %	10,0 %

In France, private medical practice operates within the framework of a regulated market. While numerous private entities hold exclusive rights to practice their skills, their activities are closely overseen and regulated by the government, particularly in pricing. The national health insurance system significantly subsidizes patient costs, covering approximately 70–80 % of baseline medical expenses, with full coverage extended to chronic illnesses such as cancer and cardiovascular disease. French authorities frequently emphasize their role as the primary employer of medical professionals. The state reimburses patients, underscoring its commitment to the principles of solidarity, universality, and healthcare accessibility, but it does not directly fund medical practitioners. This nuance is subtle yet crucial.

Hence, medical *Monopoly* in France resembles an idyllic board game where everyone emerges victorious: patients receive reimbursement for visits and care, while physicians operate in a market underpinned by state support through patient reimbursements, alleviating concerns about financial matters and access. The centralized reimbursement model enables the government to negotiate prices with healthcare providers, thereby tightening healthcare costs. Unfortunately, several penalty and strategy cards have been picked from each side lately.

## 4 The supply and demand dynamics

Healthcare demand is surging while available resources dwindle, despite repeated assurances from French institutions that the system remains sufficient. This imbalance is driven by several factors: an aging population (21.5 % of the French population is 65 or older, and 10.4 % are 75 or older) [4]; rising cancer incidence (an estimated 433.136 new cases in 2023 with an annual increase of 0.9 % for women and 0.3 % for men since 1990) [5]; and the growing prevalence of cardiovascular disease and obesity-related complications [6].

The recent “uberization” of healthcare has enabled patients to schedule appointments via smartphone, providing instant access to a wide array of specialists. Doctolib®, the leading French e-appointment platform, facilitated immediate booking for 200 million visits with 70.000 healthcare professionals in France in 2023 [7]. Tackling medical deserts has become the alpha and omega of the French government's strategy to improve healthcare access. The urban-rural paradox is more pronounced than ever. If we were to keep one book depicting the urban-rural paradox in France, it would surely be “Madame Bovary” by Gustave Flaubert. Published in 1857, the novel tells the story of Emma Bovary, a farmer's daughter who marries a country doctor, Charles Bovary, but finds rural life stifling and romanticizes city life. In 2024, Emma and Charles likely would never meet. The widening territorial disparities in access to healthcare, with a stark urban-rural gradient and the withdrawal of most medical specialties from peripheral regions, profoundly impact both access to and the trajectory of healthcare in France [6].

The supply of doctors in France is dwindling. Official communications reveal a stagnant demographic, with 197.417 active physicians—a negligible decrease of 1.3 % from 2010 to 2023 [8]. Closer examination shows that approximately 25 % of clinicians are aged 60 or older, indicating a substantial challenge for the next decade as new entrants will not fully replace retirees. The category of “active retired physicians,” which includes doctors who receive a pension but continue to practice, accounts for 9.6 % of the workforce. The private sector has seen a marked decline in appeal since 2010, with a 16.9 % drop in general medicine practitioners, reflecting a significant reduction in the number of primary care physicians. Similarly, specialists have been affected, with the median waiting time to consult a cardiologist increasing to 42 days, a 27 % rise since 2021 [7]. An aging population, the rising prevalence of chronic diseases, and an imbalanced age distribution among physicians all point to a likely surge in healthcare demand in the coming years, as Generations X and Y begin to take on leadership roles in a system already strained by demographic pressures and workforce shortages.

## 5 X and Y takes the reins: how generation X and Y expectations shape medical practice

The landscape of work-life balance for physicians has undergone subtle shifts over time. Changes in societal norms and evolutions in work culture are leaving their mark on the medical profession as well. The once customary 24- to 30-h hospital shifts have given way to 12-h shifts, primarily for safety reasons. What was once regarded in earlier eras as a testament to tireless dedication and a path to becoming a superior practitioner now contends with the relentless march of technological progress across all sectors, as well as the increasing considerations of medical-legal complexities in the workplace. Far from the solitary practice of yesterday, managing a private medical practice now entails a complex web of responsibilities. This includes recruiting assistants, making substantial investments in medical equipment, and grappling with soaring real estate prices in urban centers. While the Generation X and Y cohorts have undeniably shaped the “business model” associated with private practice, the Generation Z cohort is now entering the residency cycle with a keen eye on specialization. The competition for coveted specialties is fierce, with limited spots available in university hospitals. After six years of medical school, students must undergo ranking exams to secure their preferred specializations. The top ten specialties are intricately linked to anticipated future income, with plastic surgery, dermatology, and ophthalmology leading the pack [9]. Peers and senior colleagues, observing the dynamism of their younger counterparts with interest, have voiced pointed critiques regarding significant inflation, the inadequacy of public sector salaries, and the growing trend toward private practice. Previously hailed as the holy grail of a medical career, academic positions in university hospitals have seen a 14.5 % decline over the past 30 years. Concurrently, there has been a 133 % rise in vacant academic lecturer roles, allowing incumbents to temporarily retain their positions while transitioning to private practice [10]. While financial considerations have always been present in healthcare to some extent, the extent to which they now shape decision-making and resource allocation within the medical environment is manifest.

## 6 Corporatization of healthcare

Economies of density applied to the private medical sector suggest that increasing the concentration of services within a particular geographic area should lead to a decrease in the average extra fees. However, this theory does not hold true for the medical sector in France for several reasons. Urbanization of the liberal sector is striking, with medical professionals congregating around large specialty units or multidisciplinary offices to pool operating expenses and ensure continuity of care. This office design is becoming the norm, resulting in oligopoly in small to mid-sized urban areas. Radiologists, medical laboratories, and ophthalmologists have been pioneers in this field.

The light side of such office design relies on accessibility and year-long continuity of care, enabling patients to access multidisciplinary practices in small clinics at one site. For partners this model eases investment in costly equipment, such as magnetic resonance imaging and scanners for radiologists for example. However, the dark side of this approach lies in payment issues for both patients and potential future partners. Uniformity in consultation visits and fees among partners often leads to the highest pricing levels. The shortage of medical professionals and increased demand further complicate this balance. Practicing alone or in small committees becomes almost impossible for certain specialties, such as radiology and biology, due to the significant investments required, thereby creating dominant positions. Investment permitted by the group further strengthens the entry ticket and marked access price for new entrants, likely making them prone to higher extra fees for patients.

The coming decade is likely to witness significant consolidation within the private healthcare sector, resulting in the dominance of a few powerful medical corporations. This trend reflects an ongoing corporatization process, characterized by the growing influence of large organizations with the capacity to leverage market power for profit maximization. For instance, in 2023, the private medical biology sector in France witnessed substantial consolidation, as six major groups command over 60 % of the market share [11]. This highly lucrative sector continues to offer substantial profit margins, reaching remarkable levels even after adjusting for the impact of the Covid-19 period (18 % profitability in 2016, rising to 32 % by 2021) [11].

Another critical facet of the system's corporatization is the increasingly dominant role of managed care organizations led by private insurance companies. Since the early 2000s, the healthcare sector has witnessed a pronounced decline in the number of healthcare organizations, with the most substantial consolidation occurring between 2001 and 2016. During this period, insurance companies experienced a nearly sixfold decrease in their numbers relative to 2001 [12]. In 2022, the supplementary health insurance market was highly concentrated, with the 20 largest organizations alone capturing over 50 % of total collected premiums. The 100 largest organizations controlled 90 % of the market, underscoring the dominant position of major players. Notably, the market share of the top 20 organizations increased by 14 % compared to 2011, the first year for which comprehensive data was available [12]. In 2022, total health insurance premiums collected amounted to €40.5 billion (excluding taxes), with €29.7 billion allocated to patient reimbursements. A Senate report indicated a significant increase in supplementary health insurance tariffs of 8.1 % in 2024, with a projected rise of approximately 6.2 % in 2025 [13]. The reimbursement of additional fees is variable and remains disproportionately concentrated: 45 % of patients receive no reimbursement for these extra costs, whereas the top 10 % incur an average of €1.440 in additional expenses. Factors such as escalating healthcare expenditures (including equipment and assistants' payroll), the absence of inflation-indexed adjustments in social security reimbursement rates, and intense real estate pressures on medical facilities collectively drive the commodification of medical services. Large entities could wield significant influence over healthcare policies, enabling them to partially obstruct public interest regulations on pricing that could impact their profit margins by driving up extra fees for patients. In 2023, 50 % of specialists imposed extra fees. This implies that the market has the potential to grow rapidly with an additional 50 % capacity. Of note, 12 % of the most economically vulnerable individuals still lack coverage from supplementary health insurance in 2024 [14].

## 7 Financialization in the French medical landscape: inceptive times, Prelude to a disruptive model ?

The increasing role of private investors, corporate entities, and profit-driven models in healthcare is now a reality in France. Once seen as either mythical or insignificant, this trend has now been acknowledged by the French Medical Council. In its plenary session on March 29, 2024, the council decided to request that the French legislator abolish the possibility of non-professional third parties holding capital in a medical practice [15]. Indeed, the process of financialization has been facilitated by regulatory changes in France (Table 4). Successive relaxations of the legal framework have allowed non-practicing investors to acquire equity in professional companies (*Sociétés d’Exercice Libéral*, SEL). These regulations now permit the opening of up to 25 % of the capital of such companies, including those of doctors and midwives, to any individual or legal entity, regardless of whether they practice within the company [16]. It is challenging to precisely assess the role financial actors play in the provision of healthcare without detailed and up-to-date data on the legal and capital structures of sometimes complex and diverse companies or groups of companies. Multiple data sources must be cross-referenced, making access to these data complex.

**Table 4:** Key regulatory reforms in France and the financialization of health care.

Year	Policy Change	Description	Impact
1990s	Creation of SELs (Professional Practice Corporations)	Let liberal professionals (doctors, lawyers, etc.) organize as corporate entities. Enabled multi-site operations. Allowed up to 25 % ownership by non-professionals.	First step toward capital investment in healthcare practices. Over time, professional tax and fiscal reforms have made corporate structures such as SELs increasingly attractive, reinforcing the shift toward business-oriented practice management
2001	Murcef Law	Introduced Article 5-1, allowing biologists to own the majority share in labs where they don't practice.	Opened the door for chain formation in medical labs and allowed financial investors to enter the sector.
2004	Activity-based costing (ABC - know as T2A in France)	Funding for hospitals based on the volume and nature of medical procedures performed, rather than a global budget + B10	Introduction of a production and price based logic for hospitals to increase the volume of profitable procedures, fostering greater competition between institutions. Financial performance, measured through ABC, becomes a key management indicator and may begin to influence both medical and investment strategies. This shift can occur at the expense of less remunerative missions such as emergency care, psychiatry, and long-term care.
2009	Hospital, Patients, Health, Territories Act (HPST)	Aimed to modernize hospitals, improve healthcare access, and reorganize care provision at regional levels.	Sought to enhance financial efficiency through hospital restructuring and regional coordination.
2010	Creation of Regional Health Agencies (Agences Régionales de Santé, ARS)	Established to unify health management at the regional level, integrating various health services to improve efficiency and cost-effectiveness.	Promoted corporatization and commercialization by restructuring public hospitals to operate like competitive enterprises, leading to disrupted practices and criticism over bureaucratic drift and economically driven decisions
2013	Ballereau Law	Reformed medical biology with quality accreditation and limit financialization.	Mandatory lab accreditation encouraged consolidation. The law failed to limit financialization because it wasn't retroactive, allowing existing financial groups to expand
2018	Ordinance on Health Centers	Made it easier to open health centers without ARS approval	Unintentionally allowed investor-led chains to grow rapidly
Social Security Financing Bills (PLFSS) Post-2020	Annual PLFSS laws	Cost-containment strategies and adjustments to reimbursement rates. Periodic reductions in fee-for-service tariffs for acts in biology, radiology, etc.	These pressures compelled healthcare providers to consolidate and seek supplementary income, through measures such as charging additional fees, attracting private equity investment, narrowing the scope of services, and prioritizing higher-paying procedures
2021 & 2023	Rist Law	Regulated and cap the fees paid for temporary doctors (often charging high rates, straining hospital budgets)	This policy served as a financial control measure. High temporary staffing costs can strain budgets and increase reliance on external financing. By capping these fees, the law aimed to curb spending
2023	Valletoux law	Tightened regulation of temporary medical work, set conditions for establishing private clinics, and introduced mechanisms for overseeing financial arrangements and ownership transparency, though their scope and enforceability remain contested	This law aimed at regulating the private, for-profit sector and potentially curbing practices associated with financialization

Official reports on the financialization of the French health sector remain limited, as many institutions with access to the necessary data—such as the Court of Auditors, various ministries, Health Insurance, fiscal administration, and the Medical Council—are only beginning to engage with this issue. In its annual report, published in July 2023 and entitled “Improving the Quality of Healthcare and Controlling Costs”, the Ministry of Health addresses the financialization of the French healthcare system in just 9 out of 333 pages [17]. A key concern with this report is its broad categorization of several terms and concepts under the heading of “Financialization of Healthcare Services”, some of which could benefit from clearer definitions. The report's application of the concept of financialization appears overly broad and imprecise, as it conflates distinct phenomena within the French healthcare sector, such as asset management, corporatization, and privatization. These processes, each with its own logic and implications, differ substantially from mere financialization. As these concepts are relatively new to the French healthcare system, enhancing the clarity, definition and factual grounding of these terms will improve future analyses. In its 60-page annual report, released in July 2024, the French Medical Council makes only two brief references to “financialization” [18]. While the report sets a goal for 2024 to “take action to combat the financialization of medicine,” it fails to provide a detailed action plan. Neither report addresses critical elements such as healthcare plans and managed care organizations administered by private insurance companies, nor the potential growing market presence of banks and bank-insurance firms - indicators of increasing financialization.

Financialization entails well-identified risks, the practical reality of which remains yet poorly documented, but it can also present certain advantages. For patients, the two main risks are reduced access to care —either through increased costs, or through restructuring that leads to the reduction or closure of certain services to enhance profitability —and a decline in the quality of care due to a focus on profitability. Recent examples in France include the Orpea group [19] and certain dental or ophthalmological health centers. The “Orpea crisis” highlighted the risks to both care quality and financial stability when healthcare facilities are managed primarily as financial assets. Orpea, a French company that had grown into one of Europe's largest nursing home chains, was exposed in 2022 for prioritizing profits at the expense of care: understaffing facilities and mistreating residents. This case illustrates how an investor-oriented model, as seen with Orpea's backing by international pension and hedge funds, can significantly erode standards of care. Following the revelations, Orpea's share price crashed by 93 % in 2022. Its aggressive, debt-fueled expansion strategy pushed the company toward insolvency, ultimately necessitating a state-led bailout in 2023. Following the Orpea scandal, authorities mandated audits of nursing homes and initiated discussions on profit caps and stricter staffing requirements in elder care facilities. Similar concerns have emerged elsewhere; in the United States, for example, private equity acquisition of hospitals has been associated with declines in patient-reported care experience, leading to reduced confidence in healthcare quality [20,21]. Reports remain limited regarding the French trend in quality evaluation related to financialization. This has prompted the Organisation for Economic Co-operation and Development (OECD) to highlight the challenges ahead in monitoring costs, quality, and outcomes in the hospital sector and across the broader healthcare system, emphasizing the need for regular performance evaluation [22].

For healthcare professionals, financialization poses a risk of diminished professional autonomy and potential conflicts between financial and professional priorities. Regulators face the challenge of significant changes in healthcare structure and representation, impacting regulatory effectiveness and dialogue Financialization does not only have potentially deleterious effects. It can accelerate the achievement of public policy objectives when these require substantial investments. Certain common goals—public policy and financial investments—can be temporarily aligned. The topic of investment in the healthcare sector in France reflects the transformation of care organization, particularly the shift towards outpatient care. Private investments address the chronic underfunding of outpatient care, a consequence of state policies favoring hospital-centric care. The lack of public investment creates opportunities for private investors in healthcare activities. This is an attractive and sometimes effective strategy, but likely to be extremely risky for public authorities. For professionals, capital influx can be perceived as a financial opportunity, both individually and collectively, especially as they approach the end of their careers and seek to ease the transition into retirement.

## 8 How far has financialization Penetrated the French health care system?

### 8.1 Rise of investor-owned health care groups

Since the 2000s, France has seen a rapid rise of investor-owned health care groups, especially in certain sectors. Four hospital groups now control about 40 % of France's private hospital sector. These include Ramsay Santé, Elsan, Vivalto, and Amalfi (Amalviva Santé), all of which have grown through leveraged acquisitions. For example, Ramsay Santé and Elsan each acquired dozens of clinics across France, aiming for economies of scale.

### 8.2 Medical biology: A flagship example

The medical biology sector has emerged as an emblematic example of financialization in health care in France. For instance, the exponential growth of the leading medical biology firm includes a revenue increase from €215 million to €1.3 billion in 2020 over four years, alongside expansion to 750 locations and employment of 850 biologists. A recent leveraged buyout of €2.8 billion was completed in early 2021. The radiology sector in France exhibits notable and consistent investments driven by technological innovation and equipment life cycles, and a trend towards consolidation among major healthcare providers. These conditions are highly conducive to future financialization. Even primary care clinics and dental centers, traditionally run by self-employed doctors or dentists, have drawn interest from investment groups looking to create chains, although this remains nascent due to regulatory barriers in France [16]. One notable trend is investors finding creative ways to enter restricted sectors: for instance, some pharmacy owners (pharmacies in France must be pharmacist-owned) have financed expansion by issuing convertible bonds to investment funds, effectively ceding some control to financiers and raising concerns about professional independence.

### 8.3 Recent high-profile financial transactions

In October 2024, the U.S.-based firm Clayton Dubilier & Rice entered exclusive negotiations to acquire a 50 % controlling stake in Sanofi's consumer healthcare business, Opella, in a deal valued at approximately €16 billion. To safeguard national interests, the French state, through Bpifrance, secured a 2 % stake, ensuring commitments to employment and domestic investment. That same year, Crédit Agricole, in collaboration with the European Investment Bank, launched a €400 million financing package aimed at supporting healthcare professionals, particularly in underserved areas. Meanwhile, in the fast-moving landscape of health startups, numerous French companies have attracted substantial venture capital investments from both national and international investors. These developments reflect a dynamic and evolving healthcare ecosystem increasingly shaped by financial actors. However, fully understanding the scope and implications of these financialization processes remains a significant challenge. The complexity stems from the intricate financial structures involved, including opaque corporate arrangements, holding companies, leveraged buyouts, and complex investment vehicles. Consequently, these transformations are often difficult to grasp, not only for the general public but also for healthcare professionals, specialists, and public policymakers alike.

### 8.4 Integration of financial capital into public health financing

Evidence points to significant financial integration in France's public health system, with financial capital playing a key role in managing debt, liquidity, and infrastructure. France established CADES (Caisse d’Amortissement de la Dette Sociale) in 1996 to refinance social security debt via market-issued securities. From 1996 to 2018, it absorbed €260.5 billion in debt, over €147 billion from healthcare, and paid €72 billion in interest and fees. Funding came largely from international investors, tying the health system to global capital markets [23]. Since 2007, ACOSS (Central Agency of Social Security Organizations, the agency managing social security cash flows) has used short-term securities for liquidity management. By 2018, 93 % of short-term needs were market-financed, with €2 trillion issued in European commercial papers, making ACOSS the top global public issuer [23]. Public hospital debt rose from €12 billion in 2003 to €30 billion in 2018, with annual interest payments doubling to €1 billion by the 2010s. Many of the loans were complex, high-risk financial structures. Finally, health institutions increasingly adopt private-sector financial strategies focused on return on investment, cost control, and benchmarking. Agencies like ARS, ACOSS and CADES created financial departments staffed by market professionals, signalling a broader systemic shift.

### 8.5 Broader context and policy implications

The financialization of France's health sector must be analysed in the context of constrained public funding and a broader shift toward neoliberal policy orientation. Although not as classically neoliberal as the USA or the UK, France represents a hybrid model, maintaining elements of a social market economy while increasingly adopting neoliberal policies particularly under specific governments and in response to mounting public debt. Market-driven approaches and fiscal austerity have led to reduced state expenditure on public health services [24], necessitating alternative financing mechanisms that often involve private capital and financial markets to sustain healthcare infrastructure and delivery. As a result, healthcare institutions have increasingly adopted financial instruments and management practices characteristic of the private sector. Recent work by Cordilha has documented this phenomenon, examining how financialization is reshaping public health systems in France and elucidating the mechanisms through which financial practices permeate healthcare institutions [23]. A causal link is clear between limited public funding and the financialization of the health sector, illustrating how fiscal constraints can drive healthcare systems toward market-based models with substantial implications for public health policies and outcomes.

## 9 Financialization in neighbouring countries and healthcare systems: trends and health impacts

Germany's health system, like France's, operates on a universal, solidarity-based social insurance model. Approximately 88 % of the population is covered by statutory health insurance via non-profit “sickness funds,” with the rest privately insured, typically higher-income individuals. Service delivery is mixed, spanning public, private non-profit, and for-profit providers. Regulated competition and decentralized, multi-payer governance reflects France's structure. Germany illustrates how financialization can advance rapidly and how policy is now reacting, through both ownership rules and financing changes, to rebalance commercial and social aims. Germany has indeed experienced significant corporatization of its hospital sector and an influx of private capital in ambulatory care. Two large corporations: Helios/Fresenius and Asklepios have expanded aggressively, acquiring many community hospitals, with consolidation often financed by debt and equity markets (Fresenius is stock-listed). The logic of these acquisitions is financial expansion: as firms like Fresenius pursue “massive investments and mergers to dominate markets and secure revenue streams” [25]. The expansion of private equity in ambulatory care has also attracted significant attention. A 2004 health reform enabled the creation of Medizinische Versorgungszentren (MVZs) which stands as multidisciplinary medical centers, which could be owned by hospital operators or physician groups. This inadvertently opened primary care and outpatient specialties to outside investors. Estimates suggest that by 2020, nearly 20 % ambulatory health care centers (750 out of 3800 MVZs) were owned or backed by private equity funds [26].

Belgium's health system is a social insurance model with universal coverage via mandatory insurance managed by non-profit sickness funds and state oversight. Funded through payroll taxes and general revenues, care is delivered by a mix of public and predominantly private non-profit hospitals, many linked to Catholic groups or mutualities. High patient choice and negotiated reimbursement rates define the system, which traditionally limited commercialization due to restricted profit-making from core services. Financialization has reshaped Belgium's elder care sector, particularly in nursing homes. Cost pressures and an aging population enabled for-profit expansion, now accounting for 32.5 % of beds, compared to 29.5 % public and 38 % non-profit. Regional disparities are striking, with for-profit ownership reaching 63 % in Brussels and 48 % in Wallonia [27] Medical laboratories in Belgium have also seen consolidation similar to France's [[28b], [28a]].

Switzerland represents a universal coverage model with a strong private sector orientation, making it somewhat unique but still comparable to France in achieving universal health care. All residents must purchase basic health insurance from competing private insurers, which are not allowed to profit from basic benefit plans. Switzerland's health system has long included financialized elements, with competing insurers operating like businesses. Corporate influence has grown, especially through mergers and acquisition with two major private hospital groups Hirslanden (owned by Mediclinic International) and Swiss Medical Network (owned by AEVIS, and listed on the SIX Swiss Exchange) now dominate the private inpatient sector. Switzerland's health real estate (hospitals, clinics, senior care facilities) has attracted investors as health-related real estate is seen as a stable asset class. One notable outcome of increased financialization and the expanding role of private, profit-oriented actors is the steady rise in health insurance premiums, which continues to challenge affordability. In 2025, Swiss premiums are projected to rise by 6 %, reinforcing concerns about Switzerland's position among the least affordable healthcare systems for patients [[28b], [28a]].

## 10 Strengthening the monitoring of financialization

Financialization raises several questions: the organization of private medical structures, the coexistence and interplay of medical and financial logics, and the regulation of these entities. The shift towards, and the manifestations of “financialization” within the French healthcare system are complex to quantify [29]. This trend is difficult to measure precisely due to a lack of detailed data and the nascent nature of the transition. There are currently very few in-depth studies with sufficient perspective to analyze this phenomenon. To understand these issues, a thorough diagnosis is required. This must be based on continuously evolving knowledge. Regulatory institutions, whether national, regional, public, or professional, must grapple with this complex phenomenon. Establishing an observatory of medical financialization, aimed at analyzing demographic trends, shareholder movements, practice modes, and pricing, would provide valuable insights. These elements are essential for creating a national and European regulatory body responsible for oversight and control. The development of experts with interdisciplinary skills, covering legal, medico-economic, macroeconomic, public health, and care organization fields, will be pivotal. Expert panels are poised to investigate several critical concepts that warrant further examination.

• Investigate how a predominantly de-commodified health system, such as France's, navigates the challenges posed by limited public funding amid a sluggish economy burdened with high levels of taxation and social contributions. This analysis should focus on the system's ability to manage increasing care demands and expenditures, and its resilience against the pressures of financialization.
• Explore the social, political, and institutional dynamics surrounding the conflict between private investment interests and medical ethics within an advanced, largely de-commodified healthcare system characterized by complex regulation.
• Assess the performance of the French healthcare system in the context of recent cost-containment policies and the rise of financialization. Upcoming reports should provide detailed indicators, including survival rates for cancer and cardiovascular diseases, infant mortality rates, life expectancy, and access to free health insurance for individuals below a certain income threshold.
• Delve into the specific French institutional, sectoral, and political factors that may contribute to the rise of the current state of financialization within the French healthcare system

## 11 Conclusion

Driven by principles of equity, universal coverage, solidarity, patient choice, and accessibility, the French healthcare model faces intense challenges. Disciples of Hippocrates increasingly face an undeniable emphasis on financial considerations. The recent shift in the French model is new, as its initial values aimed to prevent or at least limit financialization. The convergence of care and finance, originally semantically opposed, is becoming increasingly intertwined. In an era of rapid and profound change for the French healthcare model, upholding ethics, accessibility, and solidarity is more critical than ever.

## Patient consent for publication

Not applicable.

## Disclosures

BM and OM declare that they have no competing interests, whether financial or non-financial, that could be perceived as relevant to the manuscript. OM reports grants in support of investigator and investigator-initiated studies from AstraZeneca, Medtronic and Boehringer Ingelheim, all outside of the submitted work. OM has been awarded grants by “Fondation Cœur et Recherche” and “Endofrance,” two reputable charities in France committed to advancing research initiatives in cardiovascular disease in endometriosis. BM declares no conflict of interest.

## Contributors

BM and OM contributed equally to the writing, editing, and substantive revision of the manuscript. Both authors had full access to all the data presented in the manuscript and accept responsibility to submit for publication.

## Ethical approval

This study did not require ethical approval as it did not involve human participants, animal subjects, or any form of sensitive data collection.

## Funding

No external funding was received for this research.

## Competing interests

The authors declare no competing interests related t this study.
